# Communication between healthcare professionals and parturients during the childbirth experience: a mixed-methods study

**DOI:** 10.1590/0034-7167-2024-0341

**Published:** 2025-08-08

**Authors:** Luciana Braz de Oliveira Paes, Natália Rejane Salim, Beatriz Rosana Gonçalves de Oliveira Toso, Jamile Claro de Castro Bussadori, Monika Wernet, Aline Oliveira Silveira, Mariana Torreglosa Ruiz, Márcia Regina Cangiani Fabbro

**Affiliations:** I Centro Universitário Padre Albino. Catanduva, São Paulo, Brazil; II Universidade Federal de São Carlos. São Carlos, São Paulo, Brazil; III Universidade Estadual do Oeste do Paraná. Cascavel, Paraná, Brazil; VI Universidade de Brasília. Brasília, Distrito Federal, Brazil; V Universidade Federal do Triângulo Mineiro. Uberaba, Minas Gerais, Brazil

**Keywords:** Parturition, Communication, Postpartum Period, Women, Personal Autonomy., Parto, Comunicación, Periodo Posparto, Mujeres, Autonomía Persona.

## Abstract

**Objectives:**

to analyze the communication between healthcare professionals and parturients during the childbirth experience from the perspective of women.

**Methods:**

this sequential explanatory mixed-methods study was conducted with postpartum women in a teaching maternity hospital. The first phase (quantitative) involved a descriptive analysis of 265 responses from the *Mother-Baby Friendly Birthing Facilities Thermometer.* In the second phase (qualitative), 44 interviews were conducted with postpartum women. Descriptive and content analysis, using a thematic approach, was applied.

**Results:**

data analysis and integration identified limitations in information transmission, a lack of understanding, and restricted autonomy in decision-making processes. Information was conveyed by the healthcare team in an authoritative manner. However, discharge instructions were well understood.

**Conclusions:**

communication between healthcare professionals and parturients during the childbirth experience is shaped by power dynamics, underscoring the need to reconsider communication as an essential component of maternal care.

## RESUMEN

**Objetivos:**

analizar la comunicación entre profesionales de la salud y parturientas durante la experiencia de parto, desde la percepción de las mujeres.

**Métodos:**

estudio mixto explicativo secuencial realizado con puérperas de una maternidad escuela. La primera etapa, cuantitativa, incluyó el análisis descriptivo de 265 respuestas del “Termómetro de la Iniciativa Hospital Amigo de la Mujer y del Niño”. En la segunda etapa, cualitativa, se realizaron 44 entrevistas con puérperas. Se utilizó análisis descriptivo y de contenido en la modalidad temática.

**Resultados:**

el análisis y la conexión de los datos identificaron limitaciones en la transmisión de información, falta de comprensión y restricción de la autonomía en los procesos de toma de decisiones. La información fue transmitida por el equipo de manera impositiva. No obstante, las orientaciones al alta fueron comprendidas.

**Conclusiones:**

la comunicación entre el equipo de salud y la parturienta durante la experiencia de parto está atravesada por relaciones de poder, lo que indica la necesidad de replantear la comunicación como un componente esencial del cuidado.

## INTRODUCTION

Effective communication is one of the pillars of recommended care practices for a positive childbirth experience, ensuring that communication between the care team and women during labor and delivery guarantees the provision of information, introductions, consent, respect, active listening, opportunities for choice, and confidentiality. In this model, it is recommended that women not only survive childbirth but also thrive and reach their full potential for health and well-being. To this end, it is essential to ensure that women give birth in an environment that is not only clinically safe but also allows them to maintain a sense of control through involvement in decision-making^(1)^.

In this context, effective communication enhances a woman’s labor experience, contributing to her autonomy and confidence, and consequently reducing the need for unnecessary interventions. Moreover, communication failures between healthcare professionals and parturients are among the leading causes of obstetric sentinel events-that is, harm caused by errors and failures in clinical practice^(2)^-as well as other adverse maternal health outcomes^(3)^.

In the context of childbirth care in Brazil, studies highlight the occurrence of uninformed or non-consensual practices, particularly at the time of delivery^(4,5)^. Although policies have been developed in recent years with a focus on the humanization of childbirth and delivery, this scenario remains challenging, as parturients do not fully have their rights guaranteed^(6)^. Far from an ideal standard of care, social determinants of health within the Brazilian context influence healthcare services, including gender, class, race, and ethnicity. Social inequalities, negligence, mistreatment, and gender-based violence contribute to practices that are demonstrably harmful to the health of women and children^(4)^, as well as to care models that neglect key aspects such as the woman’s experience during childbirth, her values, her culture, and the support network of those involved^(7)^.

Given this scenario, there is a need to promote knowledge about communication in the context of labor and childbirth in Brazil^(8)^ as a necessary step toward improving obstetric care in maternity services. Parturients and their families often lack opportunities to express their concerns regarding the care provided within healthcare services, in a model of obstetric care that should centrally strengthen the role of those experiencing childbirth^(9)^. Thus, this study underscores the importance of communication between healthcare professionals and parturients during the childbirth experience, based on a mixed-methods study, posing the question: “Does communication between professionals and parturients influence the childbirth experience from the woman’s perspective?”.

## OBJECTIVES

To analyze communication between healthcare professionals and parturients during the childbirth experience from the perspective of women.

## METHODS

### Study Design

This is a mixed-methods study with a sequential explanatory design (QUAN → qual), in which both approaches were integrated through connection. Mixed methods are defined as a research procedure that involves the collection, analysis, and combination of quantitative and qualitative techniques in a single study, justified by the premise that their interaction provides enhanced analytical possibilities^(10)^. The QUANT phase employed a cross-sectional design, while the qual phase was descriptive, structured according to the Consolidated Criteria for Reporting Qualitative Research^(11)^.

The study followed the World Health Organization (WHO) Recommendations for Intrapartum Care for a Positive Childbirth Experience in data analysis. These guidelines recognize the experience of care as a critical aspect for ensuring high-quality childbirth assistance and better woman-centered outcomes, adopting a holistic and human rights-based approach^(1)^.

### Study Setting

The research was conducted in a mediumto high-complexity teaching maternity hospital located in the countryside of São Paulo (SP), Brazil, serving as a referral center for nineteen municipalities. The site was selected for convenience, based on the researcher’s familiarity with the field and accessibility. The hospital is characterized as an obstetric center with a multidisciplinary team. However, labor and delivery are conducted by a medical team (physicians and medical students), referred to in this article as professionals, according to the terminology used in the quantitative phase instrument.

### Study Period

The first phase (QUANT) was conducted from January to June 2021, while the second phase (qual) took place from July to September 2021, occurring between one and six months postpartum. The interval between phases was necessary for the analysis of the QUANT phase data.

### Participants

The study population comprised postpartum women who gave birth at the hospital, with a total sample of 265 women.

### Selection Criteria

In the first phase (QUANT), inclusion criteria encompassed postpartum women, whether following vaginal delivery or cesarean section, who were in rooming-in care and had given birth within the last 24 hours in a hospital setting. To comply with the six-month period defined in the sample size calculation and to ensure randomization, 45 interviews per month were conducted, averaging 11 interviews per week. The interview day was chosen based on convenience.

Exclusion criteria for this phase included: Women who experienced miscarriage; Women who gave birth en route to the hospital; Women transferred from another institution; Women who required readmission; Unemancipated adolescents without the presence of a legal guardian; Postpartum women with hearing, visual, or cognitive impairments.

In the second phase (qual), inclusion criteria required prior participation in the first phase and a willingness to share their childbirth experience. In sequential explanatory mixed-methods studies, the qualitative sample must include individuals from the initial quantitative sample, as the intent is to further explore the quantitative findings^(12)^.

Exclusion criteria for this phase included women who did not have internet access. Participants were considered lost to follow-up if they: Did not participate in the second phase of instrument application (post-discharge). Did not respond to the researcher’s contact after three phone calls at different times. There were 28 cases of loss to follow-up and one maternal death, totaling 29 losses. There were no refusals in either phase of the study.

### Sample Definition

A total of 265 postpartum women participated in the QUANT phase. The precision was calculated using a two-tailed hypothesis test for prevalence estimation $\left\{ \begin{array}{l} H_{0}:p=p_{0}\\ H_{1}:p\neq p_{0} \end{array}\right.$ , where the absolute allowable estimation error (margin of error) was based on a 5% significance level: $\varepsilon =1,96\sqrt{\frac{p_{0}\left(1-p_{0}\right)}{n}}$ ^(13)^. The sample size calculation was based on the number of births in the last six months, with a monthly average of 130 deliveries per month at the institution.

The qual phase sample was drawn from the 236 postpartum women who responded to the post-discharge questions (conducted 10 days postpartum via telephone) from the first, quantitative phase of the study. Of the 236 women who agreed to participate in the second phase, a nominal list was created, and each participant was assigned a number. The interviews were conducted through manual random selection, following the order of post-discharge interviews. Theoretical saturation was reached with 44 women.

### Study Variables

Data were initially obtained through the Mother-Baby Friendly Birthing Facilities Thermometer (MBFBF-T)^(14)^, and the following specific questions were used as they relate to communication between the woman and the healthcare professional: Delivery of the birth plan; The professional requesting permission/authorization to perform a cesarean section; The professional who assisted during labor/cesarean section introduced themselves; For women who underwent induction, the professional requested permission/authorization to induce labor; Women understood the reason for labor induction; The woman was asked for authorization to perform a vaginal examination; All professionals who performed an examination informed the woman about the progression of labor; The professional requested permission to rupture the amniotic sac; The woman did not understand why the sac needed to be ruptured; The professional assisting labor introduced themselves; Women were advised to return to the hospital in case of complications. These variables address the objective of this article.

### Data Collection Instruments

The MBFBF-T questionnaire was applied to the women, along with a form for collecting sociodemographic and obstetric data, obtained from medical records and/or the participant’s prenatal card.

Originating from the National Health Service (NHS) in England^(15)^ and the Maternity Safety Thermometer (MST)^(16)^, the MBFBF-T consists of 69 questions divided into three sections (admission, hospitalization, and post-discharge). It assesses the quality of care provided, considering the practices adopted by maternity professionals, patient outcomes, and the woman’s experience regarding the care received. This includes being informed, having opportunities for decision-making, receiving respectful care, and being heard by professionals^(14)^.

The number of completed questions varied according to the type of delivery. The questionnaire contains dichotomous and multiple-choice questions, allowing women to select all applicable responses.

For the qual phase, an open-ended interview was conducted, based on observations from the quantitative phase results, aiming for a deeper understanding^(12)^. The trigger question used was: *“Tell me about the conversation between you and the professionals during your childbirth experience”.*

### Data Collection

Data from the QUANT phase, related to admission and hospitalization, were collected through face-to-face interviews at the hospital, in a private room in the maternity ward, conducted by the researcher, the first author of this manuscript. Post-discharge data were collected 10 days postpartum via telephone (postpartum-related questions). Data collection occurred over six months.

It is important to mention that, prior to data collection, a pilot study was conducted with a sample of 10 postpartum women who were not included in this research. This pilot study allowed for the organization of a private space for administering the questionnaire.

Interviews for the qual phase were conducted remotely due to the COVID-19 pandemic, using the Google Meet^®^ video calling platform, with audio recording and a duration of 30 to 40 minutes. The interviews were conducted by the principal researcher, who was previously trained to conduct them, and were scheduled according to the availability of participants via WhatsApp^®^ contact.

### Data Processing and Analysis

Data from the QUANT phase were stored in an Excel^®^ spreadsheet and analyzed using descriptive statistics, based on absolute frequencies and percentages^(17)^. The description of the responses considered whether or not they met WHO recommendations, according to the majority of responses.

The analysis of qual phase data was conducted using the Content Analysis technique, in the thematic modality. The analysis was performed in three stages: a) Pre-analysis, b) Material exploration, c) Processing and interpretation of results^(18)^.

This process involved floating reading to grasp ideas, concepts, and themes that defined the registration unit and context unit, as well as the selection of excerpts compatible with categorization and coding. Coding was performed based on phrases and words related to communication during childbirth, which were highlighted in the transcribed text and selected from the interviews. These phrases or words were regrouped multiple times as needed until they resulted in thematic categories.

After conducting separate analyses of the quantitative and qualitative approaches, the data were integrated by connection^(12)^. To organize the analysis and generate new insights^(19)^, the joint display method was used^(20)^, linking quantitative results with the highest and lowest scores to the qualitative results.

The study was approved by the Ethics Committee, obtaining the Certificate of Presentation for Ethical Appreciation, in compliance with national guidelines for human research (Resolutions No. 466/2012 and No. 510/2016 of the National Health Council). The Informed Consent Form was signed by the women and the researcher.

This article is derived from the doctoral dissertation “*Experiência positiva de parto: fatores determinantes e influenciadores na perspectiva de mulheres*”, available in the repository of the Federal University of São Carlos^(21)^.

## RESULTS

Of the 265 postpartum women, the majority had a partner (242; 91.32%), identified as white (135; 50.94%), had nine to eleven years of education (127; 47.92%), and followed the Catholic religion (130; 49.06%). Most had attended prenatal care (261; 98.49%), with a minimum of two consultations and a maximum of sixteen. The majority of women were classified as low-risk pregnancies (211; 79.63%).

Table 1 presents the variables that most closely aligned with the recommendations and those that did not meet WHO guidelines regarding effective communication.

**Table 1:** Percentage of variables associated with WHO recommendations, Catanduva, São Paulo, Brazil, 2024

Variable	Total cases	Percentage
04. Birth plan		
Not recommended response	265	100
25. Did the professional ask for your permission/authorization for a cesarean section?		
Not recommended response	71	43.29
Recommended response	93	56.71
26. Did you understand why forceps, a vacuum extractor, or a cesarean section was performed?		
Not recommended response	26	15.85
Recommended response	138	84.15
27. Did the professional who attended your delivery/cesarean section introduce themselves?		
Not recommended response	183	69.06
Recommended response	82	30.94
31. Did the professional ask for your permission/authorization to induce your labor?^*^		
Not recommended response	15	45.45
Recommended response	18	54.55
32. Did you understand the reason for the induction?		
Not recommended response	14	42.42
Recommended response	19	57.58
40. Did all professionals ask for your permission/authorization before performing vaginal examinations?		
Not recommended response	24	22.02
Recommended response	85	77.98
41. After the vaginal examination, did all professionals who examined you explain what was happening to you or how your labor was progressing? ^**^		
Not recommended response	19	17.59
Recommended response	89	82.41
43. Did the professional ask for your permission to rupture your amniotic sac?		
Not recommended response	27	64.29
Recommended response	15	35.71
44. Did you understand why your amniotic sac needed to be ruptured?		
Not recommended response	27	64.29
Recommended response	15	35.71
45.Did the professional who attended your labor introduce themselves?		
Not recommended response	75	71.43
Recommended response	30	28.57
61. Were you instructed to return to the hospital if you experienced any of the following danger signs: bleeding, severe abdominal pain, headache, seeing black spots or bright lights, stomach pain, difficulty breathing, fever or chills, cesarean section scar or vaginal stitches with purulent discharge, and/or difficulty emptying your bladder?		
Not recommended response	106	44.92
Recommended response	130	55.08

* Of the women who underwent induction;

** Of the women who went through labor.

Table 1 identifies that parturients were attended to in accordance with WHO recommendations for the following variables: the professional requested permission to perform the cesarean section (93; 56.71%), and the woman understood the reason (138; 84.15%). Of the women who underwent induction, the professional requested permission/authorization (18; 54.55%), and they understood the reason labor needed to be induced (19; 57.58%). After the vaginal examination, all professionals who performed the examination informed the women about the progression of labor (89; 82.41%), and the women were advised to return to the hospital in case of complications (130; 55.08%).

The questions that presented not recommended responses were: the absence of birth plan delivery (100%), the professional who attended labor did not introduce themselves (75; 71.43%), and the professional who assisted in the delivery/cesarean section also did not introduce themselves (183; 69.06%). The professional did not request permission to rupture the amniotic sac (27; 64.29%), and the woman did not understand why the sac needed to be ruptured (27; 64.29%).

In the qual phase, 44 postpartum women were interviewed, of whom 24 underwent cesarean sections and 20 had vaginal deliveries, with ages ranging from 21 to 31 years (25; 57%). The majority had a partner (41; 93.2%) and identified as white (24; 55%), mixed race (14; 33%), and Black (6; 12%). Regarding education, 22 (50%) had between nine and eleven years of schooling. Most were Catholic (19; 43.2%). All had attended prenatal care (44; 100%), with low-risk pregnancies predominating (211; 79.63%).

The qualitative data analysis resulted in two thematic categories. The first, titled “Ineffective communication for a positive childbirth experience”, revealed that the women were unaware of the birth plan and did not know who the professionals providing care were, yet they considered it important and valued knowing them. The lack of communication regarding what was happening to them and their babies, the absence of explanations about the indication for cesarean sections, and the lack of consent for procedures were some of the aspects that weakened their experience.

The second category, “Effective communication for a positive childbirth experience”, describes that the best experiences were related to protocol-based discharge instructions.

Chart 1 presents the integrated analysis, resulting from the connection between quantitative and qualitative findings, understood as ineffective communication for a positive childbirth experience.

**Chart 1:** Integrated analysis: ineffective communication for a positive childbirth experience, Catanduva, São Paulo, Brazil, 2024

T-IAMC With not recommended responses	Quantitative results	Qualitative results
Did not know about the birth plan	265; 100%	*I didn’t know there was a birth plan, I found out during the interview you did with me. To have the baby, they were the ones deciding what was best. Regarding the birth plan, I was never informed, not even at the health center. I didn’t know that anesthesia could be used in normal delivery.* (E12)
The professional did not ask for permission to rupture the amniotic sac	27; 64.29%	*The doctor was the one who ruptured my sac. She told me, ‘I’m going to rupture it now, okay?’ I was already at the final stage, she informed me, and I think it was to speed things up.* (E12)
The woman did not understand why the amniotic sac needed to be ruptured	27; 64.29%	*They were the ones who ruptured my sac. I already had 9 cm of dilation, I think that was why.* (E23)

Next, in Chart 2, the quantitative results are presented in connection with the qualitative results, understood as effective communication for a positive childbirth experience.

**Chart 2:** Integrated analysis: effective communication for a positive childbirth experience, Catanduva, São Paulo, Brazil, 2024

T-IAMC With recommended responses	Quantitative results	Qualitative results
Guidance on returning to the hospital if presenting any signs of danger or puerperal infection	130; 55.08%	*Before discharge, I received some guidance from the nurses and doctors. They advised me to go to the health center for a follow-up to remove the stitches. So far, I haven’t had any problems, the stitches didn’t get infected, everything is fine.* (E14)

The following presents contradictory data regarding effective communication during the childbirth process in the connection between quantitative and qualitative data, where qualitative results reveal barriers in communication with the healthcare team. Participants reported imposed forms of communication with little to no room for dialogue (Chart 3).

**Chart 3:** Integrated analysis: contradictory variables in the connection between qualitative and quantitative data for effective communication in a positive childbirth experience, Catanduva, São Paulo, Brazil, 2024

T-IAMC With recommended responses	Quantitative results	Qualitative results
The woman understood the reason for the cesarean section	138; 84.15%	*We have to accept how things work; everything is already routine. They decide how the delivery will be, whether it will be vaginal or cesarean. We don’t have the freedom to discuss it. If we speak up, they don’t like it, and we don’t have many rights. We arrive without knowing who we will consult with or what will happen.* (E29)
After the vaginal examination, professionals informed the woman about labor progression	89; 82.41%	[...] *some would mention how much dilation there was, others wouldn’t.* (E29)
Professionals requested permission/authorization to perform the vaginal examination	85; 77.98%	*They already come in with gloves on; it’s imposed.* (E36) *The vaginal exams are not consented to; they are only announced. They come in and say they need to do it, but they don’t ask. We end up not saying anything-how am I supposed to say no, that I don’t want it!?* (E42)
The woman understood the reason for labor induction	19; 57.58%	*This labor was induced with misoprostol, and later they started an IV drip. Everything was very forced and painful. Induction was a negative experience-many hours of labor, a lot of pain. If I had known, I wouldn’t have wanted a delivery like this* [...]. (E40)
The professional requested permission to perform the cesarean section	93; 56.71%	*They would say they were going to do it, but they didn’t ask for authorization to perform the cesarean section, just informed that they would do it.* (E5)
The professional requested permission/authorization to induce labor	18; 54.55%	*I arrived there with no dilation and low amniotic fluid, and they asked if they could induce labor. I agreed, but I didn’t fully consent, yet they induced it anyway.* (E19)

## DISCUSSION

The integrated results highlight the insufficiency and limitations of communication between parturients and healthcare professionals. These communication failures appear to begin as early as the prenatal stage, a crucial moment for birth preparation and discussion of the birth plan. The lack of communication regarding birth preparation weakens decision-making and the exercise of autonomy during labor. Women are deprived of essential information during prenatal care, and even in the maternity setting, guidance is hierarchical and limited, failing to support their participation in decision-making^(22)^.

Among the aspects of ineffective communication during the childbirth experience observed in this study, the lack of knowledge about or implementation of the birth plan (265; 100%) stands out, as confirmed in the qualitative phase, as well as the absence of information or preparation for childbirth. The little information provided was not well understood, and there was no sharing of scientific evidence, which in some cases encouraged cesarean section. Furthermore, even when a birth plan was developed, it had a low rate of adherence^(23)^. This underscores the need for public policies that train and encourage healthcare professionals to use this tool, promoting women’s autonomy^(24)^ while also improving its implementation and adherence^(23-25)^.

The integrated results also showed that discharge instructions were protocol-based and, despite offering little room for dialogue, were understood by women and interpreted as a form of effective communication. However, it is necessary to update practices and challenge the power and authority structures that arise from the institutionalization of hospital routines and standardized, hierarchical care, which pose a risk to perinatal health^(6,26)^. The nursing team is one of the most involved in post-discharge guidance, and despite efforts to facilitate the transition of care for postpartum women through education and counseling at discharge, further management strategies are needed to implement systematic actions that ensure continuity of care^(27)^, value the individuality of postpartum women, and are provided in a timely and comprehensive manner^(28)^.

Some aspects diverged in the integrated analysis, demonstrating a strength of the mixed-methods study. Some quantitative findings, initially interpreted as positive, differed from the qualitative results. During interviews, women explained that their understanding of or consent for procedures was imposed upon them-meaning they were only informed and had no opportunity to participate in decision-making. They reported not knowing what was happening to them, being unable to refuse procedures, and not understanding the reason for the cesarean section recommendation. These aspects weakened the positive childbirth experience by violating the recommendation for effective communication between maternity care providers and women.

Corroborating evidence of ineffective communication, reports of mistreatment during childbirth are increasing, often through non-consensual interventions or informal coercion- a violation linked to the deprivation of autonomy, a central principle in medical ethics and healthcare^(29,30)^, which constitutes a form of obstetric violence^(31)^. To achieve improvements in maternal and neonatal health and ensure the exercise of autonomy, communication must allow women to participate in decision-making freely and consciously^(32)^. It is important to emphasize that a parturient’s decision-making depends on balanced, complete, and unbiased information, requiring greater attention from healthcare professionals to identify the woman’s needs^(33)^. In Brazil, although childbirth humanization policies emphasize female autonomy, restrictions on women’s decision-making power in childbirth remain evident due to biopower, disciplinary power, and professional knowledge-power dynamics^(34)^.

Persuasive communication highlights the complexity of power relations in obstetric care, which is also reproduced in the field of education. The neglect of women’s decision-making power during childbirth signals the need to address women’s human rights, strengthening them through the involvement of social actors in education, management, and care^(34)^. It is essential to enhance the development of communication skills to improve the quality of obstetric care^(8)^. Therefore, it is necessary to revise training models in healthcare to promote change in the obstetric landscape, moving away from institutionalized practices and prioritizing education based on scientific knowledge that positions women as protagonists^(24)^.

Ensuring women’s autonomy through effective communication guarantees a positive childbirth experience^(35)^. In this regard, this study highlights the need to strengthen effective communication between healthcare professionals and women during labor. It is essential to engage professionals in discussions about coercion, persuasion, and women’s rights in childbirth. Public policies can, in fact, be successfully implemented when teams engage in discussions about their perceptions and the challenges they face in practicing communication based on the human right to be informed clearly and truthfully^(8)^.

Aligned with the perspective of an effective communication model, nursing plays a crucial role in redesigning obstetric care by promoting evidence-based humanization^(36)^. Through effective communication, nurses empower women during childbirth^(8)^. Their role at this moment contributes to humanization, improves the quality of obstetric care, and enhances both maternal satisfaction and safety. Additionally, an increasing number of studies highlight the value that parturients place on nurses’ involvement^(37,38)^, promoting greater well-being for both mother and baby, reducing unnecessary interventions, and increasing the number of vaginal deliveries^(37)^. This situation underscores the significant impact nurses can have in transforming harmful practices, as their role in the birthing process fosters higher-quality care and challenges the dominant obstetric model^(39)^.

### Study limitations

The exploration of communication guided by the MBFBF-T was a limitation of this study due to the absence of a scoring system in this instrument. The presence of a scoring system would have allowed for the conversion of qualitative data into quantitative data, enabling a metric for comparison and facilitating data integration. However, the use of both approaches in this study helped overcome potential limitations, as the weaknesses of one method were compensated by the strengths of the other, providing a broader perspective through the mixed-methods approach. Additionally, the fact that quantitative data were collected while the women were still in the maternity ward may have influenced their responses, as they were under the care of the healthcare team.

### Contributions to the Field of Nursing

This article contributes to nursing and interdisciplinary teams involved in obstetric care by highlighting the fragility of communication throughout the pregnancy-puerperal cycle. In doing so, it reinforces the need for a horizontalized care approach that allows for greater autonomy for women. In this context, the study may support public policies aimed at the effective implementation of instruments that promote women’s decision-making power, such as the birth plan. Furthermore, this study is expected to encourage healthcare teams to assess effective communication in maternity settings as a fundamental requirement for quality care, whether through specific instruments or internal audits, ensuring that parturients have this right guaranteed.

## CONCLUSIONS

This study allowed for a better understanding of how communication between healthcare professionals and parturients during the childbirth experience involves a power dynamic in which information is imposed, offering limited opportunities for women’s participation. This highlights the asymmetry of power between professionals, particularly physicians, and parturients in negotiations, decision-making, and care practices in the childbirth setting. Women do not receive adequate and high-quality information, either during prenatal care or labor, and the birth plan is often unknown to them.

The integrated analysis revealed that procedures are imposed and not well understood, with women merely being informed of the professional’s decision, which violates the recommendations for effective communication as outlined in intrapartum guidelines for a positive childbirth experience. Discharge instructions are provided in a protocol-based manner, and while they are understood by the women and considered effective, the key reflection proposed here is whether there is, in fact, a sensitive, empathetic, respectful, and welcoming approach to listening.

## Data Availability

The contents underlying the research text are contained in the manuscript.
