# Supplementary material for: Communication between healthcare professionals and parturients during the childbirth experience: a mixed-methods study
Source: Rev Bras Enferm. 2025 Aug 8;78(3):e20240341. doi: 10.1590/0034-7167-2024-0341 (PMC12334199; doi:10.1590/0034-7167-2024-0341)
Supplement: Translation

# Translation: Comunicação entre profissionais de saúde e parturientes durante a experiência de parto: um estudo misto

**DOI:** 10.1590/0034-7167-2024-0341pt

**Authors:** Luciana Braz de Oliveira Paes, Natália Rejane Salim, Beatriz Rosana Gonçalves de Oliveira Toso, Jamile Claro de Castro Bussadori, Monika Wernet, Aline Oliveira Silveira, Mariana Torreglosa Ruiz, Márcia Regina Cangiani Fabbro

**Affiliations:** I Centro Universitário Padre Albino. Catanduva, São Paulo, Brasil; II Universidade Federal de São Carlos. São Carlos, São Paulo, Brasil; III Universidade Estadual do Oeste do Paraná. Cascavel, Paraná, Brasil; VI Universidade de Brasília. Brasília, Distrito Federal, Brasil; V Universidade Federal do Triângulo Mineiro. Uberaba, Minas Gerais, Brasil

**Keywords:** Parto, Comunicação, Período Pós-Parto, Mulheres, Autonomia Pessoal.

## Abstract

**Objetivos:**

analisar a comunicação entre profissionais de saúde e parturientes durante a experiência de parto, a partir da percepção das mulheres.

**Métodos:**

estudo misto explanatório sequencial realizado com puérperas de uma maternidade-escola. A primeira etapa, quantitativa, envolveu a análise descritiva de 265 respostas do “Termômetro da Iniciativa Hospital Amigo da Mulher e da Criança”. Na segunda etapa, qualitativa, foram realizadas 44 entrevistas com puérperas. Utilizou-se análise descritiva e de conteúdo, na modalidade temática.

**Resultados:**

a análise e conexão dos dados identificaram limitações na transmissão de informações, ausência de entendimento e restrição da autonomia nos processos de decisão. As informações foram transmitidas pela equipe de forma impositiva. No entanto, as orientações de alta foram compreendidas.

**Conclusões:**

a comunicação entre a equipe de saúde e a parturiente durante a experiência de parto é atravessada por relações de poder, indicando a necessidade de repensar a comunicação como um componente essencial do cuidado.

## INTRODUÇÃO

A comunicação efetiva compõe um dos pilares das recomendações de práticas de cuidado para uma experiência positiva de parto, contemplando uma comunicação entre a equipe de cuidados e as mulheres durante o trabalho de parto e o parto que garanta informação, apresentação, consentimento, respeito, escuta ativa, oportunidade de escolha e confidencialidade. Nesse modelo, recomenda-se que as mulheres não apenas sobrevivam ao parto, mas também que prosperem e alcancem seu pleno potencial de saúde e vida. Para tanto, valoriza-se a garantia de que as mulheres deem à luz em um ambiente que, além de estar seguro do ponto de vista clínico, permita que elas tenham um senso de controle por meio do envolvimento na tomada de decisões^(1)^.

Nesse contexto, a comunicação eficaz melhora a experiência da mulher no trabalho de parto, contribuindo para sua autonomia e confiança e, consequentemente, reduzindo a necessidade de intervenções desnecessárias. Ademais, falhas de comunicação entre profissionais de saúde e parturientes são uma das principais causas de eventos sentinela obstétricos, ou seja, danos causados devido a erros e falhas na prática assistencial^(2)^, além de outros resultados adversos nos cuidados maternos^(3)^.

Na assistência ao parto no Brasil, estudos reforçam a ocorrência de práticas não informadas ou não consentidas, principalmente no momento do parto^(4,5)^. Por mais que, nos últimos anos, políticas tenham sido desenvolvidas com foco na humanização do parto e nascimento, esse cenário ainda se mantém desafiador, considerando que as parturientes não têm seus direitos plenamente assegurados^(6)^. Distante de uma assistência ideal, determinantes sociais da saúde, presentes no contexto brasileiro, atravessam os serviços de saúde, como gênero, classe, raça e etnia. As desigualdades sociais, a negligência, os maus-tratos e a violência baseada em gênero contribuem para práticas comprovadamente prejudiciais à saúde das mulheres e das crianças^(4)^, bem como para uma assistência que negligencia aspectos como a experiência da mulher durante o parto, seus valores, sua cultura e a rede de apoio das pessoas envolvidas^(7)^.

Diante desse cenário, há necessidade de promover o conhecimento sobre a comunicação no contexto do trabalho de parto e nascimento no Brasil^(8)^, como requisito para a melhoria da atenção obstétrica nos serviços de parto e nascimento. Parturientes e familiares carecem de serem ouvidos em seus anseios no que tange aos cuidados ofertados no âmbito dos serviços de saúde, em um modelo obstétrico que fortaleça o protagonismo das pessoas em processo de nascimento de forma central^(9)^. Assim, este estudo remete à importância da comunicação entre profissionais e parturientes durante a experiência de parto, a partir de um estudo de delineamento misto, sob a pergunta: “A comunicação entre profissionais e parturientes interfere na experiência de parto sob a percepção da mulher?”.

## OBJETIVOS

Analisar a comunicação entre profissionais e parturientes durante a experiência de parto, mediante a percepção da mulher.

## MÉTODOS

### Delineamento do estudo

Trata-se de um estudo de método misto, com delineamento explanatório sequencial (QUAN → qual), ambas as abordagens combinadas por conexão. Métodos mistos são definidos como um procedimento de coleta, análise e combinação de técnicas quantitativas e qualitativas em uma mesma pesquisa, justificado por apresentar uma abordagem em que a interação entre elas fornece melhores possibilidades analíticas^(10)^. A etapa QUANT utilizou o delineamento transversal, e a *qual* se caracterizou como descritiva, apresentada abaixo a partir do *Consolidated criteria for reporting qualitative research* ^(11)^.

Foram utilizadas as diretrizes das “Recomendações da OMS para cuidados intraparto para experiência positiva de parto” na análise dos dados. Essas recomendações concebem a experiência do cuidado como um aspecto crítico para garantir uma assistência de parto de alta qualidade e melhores resultados centrados na parturiente, com uma abordagem holística baseada em direitos humanos^(1)^.

### Local do estudo

A pesquisa foi realizada em uma maternidade escola de média e alta complexidade, localizada no interior paulista (SP), Brasil, referência para dezenove municípios. A escolha do local se deu por conveniência, relacionada ao reconhecimento do campo e das vias de acesso pela pesquisadora principal. O local caracteriza-se por ser um centro obstétrico, composto por equipe multiprofissional. Contudo, o trabalho de parto e o parto são conduzidos por equipe médica (médicos e estudantes de medicina), mencionados neste artigo como profissionais, conforme nomeado pelo instrumento da etapa quantitativa.

### Período

A primeira etapa (QUANT) foi realizada de janeiro a junho de 2021, e a segunda etapa (qual), de julho a setembro de 2021, entre um e seis meses após o parto. O intervalo entre as etapas foi necessário para a análise da etapa QUANT.

### Participantes

A população do estudo correspondeu às puérperas que tiveram seus filhos neste hospital, sendo a amostra constituída por 265 mulheres.

### Critérios de seleção

Na primeira etapa (QUANT), foram incluídas puérperas no pós-parto normal ou pós-cesariana em alojamento conjunto, que haviam parido nas últimas 24 horas em ambiente hospitalar. Para respeitar o período de seis meses do critério de cálculo amostral e garantir a aleatorização, foram realizadas 45 entrevistas por mês, 11 por semana. A escolha do dia da semana se deu por conveniência.

Não foram incluídas nesta etapa as mulheres que passaram por situações como: abortamento; parto ocorrido no trajeto para o hospital; transferência de outra instituição; reinternações; adolescentes não emancipadas sem a presença do(a) responsável legal; puérperas com deficiência auditiva, visual ou cognitiva.

Na segunda etapa (qual), o critério de inclusão foi a participação na primeira etapa do estudo e a disposição para relatar sua experiência de parto. Nos métodos mistos do tipo sequencial explanatório, a amostra qualitativa deve conter indivíduos que fazem parte da amostra quantitativa inicial, pois a intenção é explorar os resultados quantitativos em maior profundidade^(12)^.

Não foram incluídas nesta etapa mulheres que não possuíam acesso à internet. Foram consideradas perdas as mulheres que não participaram da segunda fase da aplicação do instrumento (pós-alta) e aquelas que não atenderam ao contato da pesquisadora em três ligações, em horários diferentes. Houve 28 perdas por seguimento e uma por óbito materno, totalizando 29 perdas. Não houve recusas nas duas etapas do estudo.

### Definição da amostra

Participaram da etapa (QUANT) 265 puérperas. A precisão foi calculada considerando um teste de hipóteses bilateral para estimativa de uma prevalência, $\left\{ \begin{array}{l} H_{0}:p=p_{0}\\ H_{1}:p\neq p_{0} \end{array}\right.$ onde o erro absoluto tolerável da estimativa (margem de erro) considerou um nível de significância de 5%: $\varepsilon =1,96\sqrt{\frac{p_{0}\left(1-p_{0}\right)}{n}}$ ^(13)^. O cálculo amostral foi realizado com base no número de nascimento nos últimos seis 6 meses, cuja média mensal na instituição foi de 130 partos/mês.

A amostra da etapa (qual) foi obtida a partir das 236 puérperas que responderam as questões pós-alta (realizada 10 dias após o parto, por telefone), da primeira etapa do estudo, quantitativa. Das 236 mulheres que se disponibilizaram a participar da segunda fase do estudo, foi elaborado uma relação nominal e, em seguida, foi atribuído um número. A entrevista foi realizada por sorteio manual, após enumerar as participantes na ordem das entrevistas pós-alta. A saturação teórica foi alcançada com 44 mulheres.

### Variáveis do estudo

Os dados foram obtidos inicialmente por meio do Termômetro da Iniciativa Hospital Amigo da Mulher e da Criança (T-IHAMC)^(14)^, sendo utilizadas as questões 4, 25, 27, 31, 32, 40, 41, 43, 44, 45 e 61, por estarem relacionadas à comunicação entre a mulher e o profissional. São elas: entrega do plano de parto; o profissional pedir permissão/autorização para realizar a cesárea; o profissional que atendeu o trabalho de parto/cesárea se apresentou; para as mulheres que tiveram indução, o profissional pediu permissão/autorização para induzir o trabalho de parto; as mulheres entenderam o motivo da indução do trabalho de parto; foi solicitado à mulher autorização para realizar o toque vaginal; todos os profissionais que examinaram informaram a evolução do trabalho de parto; o profissional pediu permissão para romper/estourar a bolsa; a mulher não entendeu o motivo pelo qual a bolsa precisou ser rompida/estourada; o profissional que assistiu ao trabalho de parto se apresentou; as mulheres foram orientadas a retornar ao hospital em caso de intercorrências. Essas variáveis respondem ao objetivo deste artigo.

### Instrumentos utilizados para a coleta das informações

Foram utilizados o questionário T-IHAMC, aplicado às mulheres, e um formulário para coleta de dados sociodemográficos e obstétricos, obtidos no prontuário e/ou no cartão de pré-natal da participante.

De origem no Serviço Nacional de Saúde Inglês^(15)^ e do *Termômetro de Segurança na Maternidade* ^(16)^, o T-IHAMC possui 69 questões distribuídas em três blocos (dados de admissão, internação e pós-alta) e avalia a qualidade da assistência oferecida, considerando as práticas adotadas pelos profissionais da maternidade, os desfechos e, também, a experiência da mulher com relação ao cuidado recebido. Isso inclui ser informada, ter oportunidade de escolha, receber cuidado respeitoso e ser ouvida pelos profissionais^(14)^. O número de perguntas preenchidas depende do tipo de parto. O questionário apresenta respostas dicotômicas e de múltipla escolha, permitindo que a mulher marque todas as opções aplicáveis.

Para a etapa qual, utilizou-se uma entrevista aberta, baseada na observação dos resultados obtidos na fase quantitativa, visando maior compreensão e aprofundamento^(12)^, com a seguinte questão desencadeadora: *“Conte-me como foi a conversa entre você e os profissionais durante sua experiência de parto”.*

### Coleta de dados

Foram coletados dados da etapa (QUANT), referentes à admissão e internação, por meio de entrevista face a face no hospital, em sala privativa da maternidade, realizada pela pesquisadora, primeira autora deste manuscrito. Os dados pós-alta foram coletados 10 dias após o parto, por telefone (questões do pós-parto). A coleta ocorreu ao longo de seis meses.

Cabe mencionar que, previamente à coleta de dados, foi realizado um estudo piloto com uma amostra de 10 puérperas não incluídas nesta pesquisa, o qual possibilitou a organização de um espaço privativo para a aplicação do questionário.

As entrevistas da etapa (qual) foram realizadas remotamente devido à pandemia da COVID-19, utilizando a plataforma de videochamada *Google Meet* ^®^, com gravação de áudio e duração de 30 a 40 minutos. As entrevistas foram conduzidas pela pesquisadora principal, previamente capacitada para realizá-las, e agendadas conforme a disponibilidade das participantes, por meio de contato via *WhatsApp* ^®^.

### Tratamento e análise dos dados

Os dados da etapa (QUANT) foram armazenados em uma planilha do *Excel* ^®^ e analisados por estatística descritiva, por meio de frequências absolutas e percentuais^(17)^. A descrição das respostas considerou se atendiam ou não às recomendações da OMS, de acordo com a maioria das respostas.

A análise dos dados da etapa (qual) foi realizada por meio da técnica de *Análise de Conteúdo*, modalidade temática. A análise foi conduzida em três etapas: a) pré-análise, b) exploração do material e c) tratamento dos resultados obtidos e interpretação^(18)^. Esse processo envolveu uma leitura flutuante para apreender ideias, conceitos e temas que determinassem a unidade de registro e a unidade de contexto, bem como a seleção de trechos compatíveis com a categorização e codificação. A codificação foi realizada a partir das frases e palavras relacionadas à comunicação durante a experiência do parto, destacadas no texto transcrito e selecionadas das entrevistas. Essas frases ou palavras foram reagrupadas quantas vezes fosse necessário até culminar nas categorias temáticas.

Após as análises individuais das abordagens quantitativa e qualitativa, os dados foram integrados por conexão^(12)^. Para organizar a análise e propiciar novas ideias^(19)^, optou-se por utilizar o *joint display* ^(20)^, conectando os resultados quantitativos que apresentaram maiores e menores escores aos resultados qualitativos.

O estudo foi aprovado pelo Comitê de Ética, obtendo o *Certificate of Presentation for Ethical Appreciation* (CAAE), em conformidade com as diretrizes nacionais para pesquisa com seres humanos (Resoluções nº 466/2012 e nº 510/2016 do Conselho Nacional de Saúde). O *Termo de Consentimento Livre e Esclarecido* foi assinado pelas mulheres e pelo pesquisador.

Este artigo deriva da tese de doutorado *“Experiência positiva de parto: fatores determinantes e influenciadores na perspectiva de mulheres”*, disponível no repositório da Universidade Federal de São Carlos^(21)^.

### RESULTADOS

Das 265 puérperas, a maioria tinha companheiro (242; 91,32%), declaravam-se de cor branca (135;50,94%), escolaridade de nove a onze anos (127;47,92%) e de religião católica (130; 49,06%). Realizaram pré-natal (261;98,49%), com o número mínimo de duas consultas e o máximo de dezesseis, a maior parte das mulheres classificavam-se como gestantes de risco habitual (211; 79,63%). A Tabela 1 demostra as variáveis que mais se aproximaram da recomendação e as que não atenderam a recomendação da OMS, quanto a comunicação efetiva.

**Tabela 1:** Percentual por variável associadas a recomendação da OMS, Catanduva, São Paulo, Brasil, 2024

Variável	Total de casos	Percentual
04. Plano de parto		
Resposta não recomendada	265	100
25. O profissional pediu sua permissão/autorização a cesárea?		
Resposta não recomendada	71	43,29
Resposta recomendada	93	56,71
26. Você entendeu por que foi feito um fórceps, um vácuo extrator ou uma cesárea?		
Resposta não recomendada	26	15,85
Resposta recomendada	138	84,15
27. O profissional que atendeu o parto/ cesárea se apresentou?		
Resposta não recomendada	183	69,06
Resposta recomendada	82	30,94
31. O profissional pediu sua permissão/autorização para induzir seu trabalho de parto? ^*^		
Resposta não recomendada	15	45,45
Resposta recomendada	18	54,55
32. Entendeu porquê da indução?		
Resposta não recomendada	14	42,42
Resposta recomendada	19	57,58
40. Todos os profissionais pediram sua permissão/autorização antes para fazer os exames de toque vaginal?		
Resposta não recomendada	24	22,02
Resposta recomendada	85	77,98
41. Após o exame de toque vaginal, todos os profissionais que te examinaram contaram o que estava acontecendo com você ou como estava a evolução do trabalho de parto? ^**^		
Resposta não recomendada	19	17,59
Resposta recomendada	89	82,41
43. O profissional pediu sua permissão para romper/estourar sua bolsa?		
Resposta não recomendada	27	64,29
Resposta recomendada	15	35,71
44. Você entendeu por que a sua bolsa precisou ser rompida/estourada?		
Resposta não recomendada	27	64,29
Resposta recomendada	15	35,71
45. O profissional que atendeu seu trabalho de parto se apresentou?		
Resposta não recomendada	75	71,43
Resposta recomendada	30	28,57
61. Você foi orientada para voltar ao hospital caso tivesse alguns desses sinais de perigo: sangramento, dor abdominal intensa, dor de cabeça, ver pontinhos pretos ou brilhantes, dor de estômago, dificuldade para respirar, febre ou calafrios, cicatriz da cesárea ou dos pontos na vagina com secreção purulenta e/ou dificuldade para esvaziar a bexiga?		
Resposta não recomendada	106	44,92
Resposta recomendada	130	55,08

* Das mulheres que passaram por indução;

** Das mulheres que que passaram por trabalho de parto.

A Tabela 1 identifica que as parturientes foram atendidas quanto às recomendações da OMS nas seguintes variáveis: o profissional pediu permissão para realizar a cesárea (93; 56,71%), e a mulher entendeu o motivo (138; 84,15%). Das mulheres que passaram por indução, o profissional pediu permissão/autorização (18; 54,55%) e elas entenderam o motivo pelo qual o trabalho de parto precisou ser induzido (19; 57,58%). Após o exame de toque vaginal, todos os profissionais que examinaram informaram a evolução do trabalho de parto (89; 82,41%), e as mulheres foram orientadas a retornar ao hospital em caso de intercorrências (130; 55,08%).

As questões que apresentaram respostas não recomendadas foram: ausência da entrega do plano de parto (100%), o profissional que atendeu o trabalho de parto não se apresentou (75; 71,43%) e o profissional que assistiu ao parto/cesárea também não se apresentou (183; 69,06%). O profissional não pediu permissão para romper a bolsa (27; 64,29%), e a mulher não entendeu o motivo pelo qual a bolsa precisou ser rompida (27; 64,29%).

Na etapa (qual), 44 puérperas foram entrevistadas, das quais 24 passaram por cesarianas e 20 por parto normal, com idade entre 21 e 31 anos (25; 57%). A maioria delas tinha companheiro (41; 93,2%), e declaravam-se de cor branca (24; 55%), parda (14; 33%) e negra (6; 12%). Quanto à escolaridade, 22 (50%) tinham entre nove e onze anos de estudo. A maioria era católica (19; 43,2%). Todas realizaram pré-natal (44; 100%), predominando gestações de risco habitual (211; 79,63%).

A análise dos dados qualitativos resultou em duas categorias temáticas. A primeira, denominada “Comunicação ineficaz para experiência positiva de parto”, revelou que as mulheres não conheciam o plano de parto, não sabiam quem eram os profissionais que estavam prestando assistência, mas consideravam importante e valorizavam conhecê-los. A falta de comunicação sobre o que estava acontecendo com elas e com seus filhos, a ausência de explicações em relação à indicação de cesárea e a falta de consentimento para a realização de procedimentos foram alguns dos aspectos que fragilizaram a experiência.

A segunda categoria, “Comunicação eficaz para experiência positiva de parto”, descreve que as melhores experiências estiveram relacionadas às orientações protocolares no momento da alta.

O Quadro 1 apresenta a análise integrada, resultado da conexão entre os resultados quantitativos e qualitativos, compreendidos como uma comunicação ineficaz para uma experiência positiva.

**Quadro 1:** Análise integrada: comunicação ineficaz para uma experiência positiva de parto, Catanduva, São Paulo, Brasil, 2024

Variável T-IAMC Com respostas não recomendadas	Resultados quantitativos	Resultados qualitativos
Não conheciam o plano de parto	265; 100%	*Eu não sabia que existia plano de parto, soube na entrevista que você fez comigo. Para ter o bebê eles que foram escolhendo o que era melhor, de plano de parto, nunca me orientaram, nem no posto de saúde. Não sabia que no parto normal pode fazer anestesia.* (E12)
O profissional não pediu permissão para romper a bolsa	27; 64,29%	*A minha bolsa foi a doutora que rompeu, ela me falou vou romper ela tá, já estava no finalzinho, ela me avisou, eu acho que é para ser mais rápido*. (E12)
A mulher não entendeu por que a bolsa precisou ser rompida	27; 64,29%	*Eles que romperam minha bolsa, já estava com 9 de dilatação, acho, foi por isso.* (E23)

Em seguida, no Quadro 2, apresentam-se os resultados quantitativos conectados aos resultados qualitativos, compreendidos como comunicação eficaz para a experiência positiva do parto.

**Quadro 2:** Análise integrada: comunicação eficaz para uma experiência positiva de parto, Catanduva, São Paulo, Brasil, 2024

Variável T-IAMC Com respostas recomendadas	Resultados quantitativos	Resultados qualitativos
Orientação de voltar ao hospital se apresentar algum sinal de perigo ou infecção puerperal	130; 55,08%	*Antes de dar alta, recebi algumas orientações das enfermeiras e dos médicos, eles me orientaram a ir consultar no postinho, para tirar os pontos. Até agora não tive problema nenhum, não infeccionou os pontos, tudo bem.* (E14)

A seguir, apresentam-se dados contraditórios para uma comunicação eficaz durante o processo de parturição na conexão de dados quantitativos e qualitativos, em que os resultados qualitativos mostram entraves na comunicação da equipe de saúde. As participantes relatam formas impositivas de comunicação e com pouco ou nenhum espaço para diálogo (Quadro 3).

**Quadro 3:** Análise integrada: variáveis contraditórias na conexão de dados qualitativos e quantitativos para a comunicação eficaz na experiência positiva do parto, Catanduva, São Paulo, Brasil, 2024

Variável T-IAMC Com respostas recomendadas	Resultados quantitativos	Resultados qualitativos
A mulher entendeu o porquê da cesárea	138; 84,15%	*Temos que aceitar como as coisas funcionam, tudo já rotina. Eles que falam que jeito será o parto, se vai ser normal ou cesárea, a gente não tem liberdade para conversar, se a gente fala, não gostam, não temos muito direitos. Chegamos sem saber com quem vamos consultar e o que vai acontecer.* (E29)
Após o toque vaginal, os profissionais contaram como estava a evolução	89; 82,41%	[...]*alguns até falava quanto estava a dilatação, outros não.* (E29)
Os profissionais pediram permissão/autorização para fazer o toque vaginal	85; 77,98%	*Eles já chegam com a luva na mão, é imposto.* (E36) *Os toques não são consentidos e sim avisados, eles chegam e falam que precisa tocar, não pedem não, e a gente acaba não falando nada, como vou falar não, não quero!?* (E42)
A mulher entendeu o motivo da indução	19; 57,58%	*Este parto foi induzido com misoprostol, colocaram soro depois de um tempo, então é tudo muito forçado, doloroso. Foi negativo a indução, muitas horas de trabalho de parto, muita dor, se eu soubesse não queria parto assim* [...]. (E40)
O profissional pediu para realizar a cesárea.	93; 56,71%	*Eles falavam que ia fazer, não pediam autorização para fazer cesárea, mas falavam que iriam fazer*. (E5)
O profissional pediu permissão/autorização para induzir o trabalho de parto.	18; 54,55%	*Também cheguei lá, não tinha dilatação e estava com pouco líquido e perguntaram se poderia induzir, eu deixei, mas não concordei muito não, mas induziu mesmo assim.* (E19)

## DISCUSSÃO

Os resultados integrados evidenciam a insuficiência e a limitação da comunicação entre parturientes e profissionais. Essas falhas na comunicação parecem ter início já no pré-natal, um momento oportuno para a preparação para o parto e a discussão sobre o plano de parto. A falta de comunicação no que tange à preparação para o parto fragiliza a tomada de decisões e o exercício da autonomia no processo de parto. As mulheres são privadas de informações essenciais durante o pré-natal e, mesmo na maternidade, as orientações são verticalizadas e limitadas, não favorecendo sua participação nas decisões^(22)^.

Dentre os aspectos da comunicação ineficaz durante a experiência de parto observados neste estudo, destacam-se a ausência do conhecimento ou da realização do plano de parto (265; 100%), referendada na etapa qualitativa, bem como a falta de informações ou preparo para o parto. As poucas informações recebidas não foram bem compreendidas e não houve compartilhamento de evidências científicas, o que, em alguns casos, incentivou a realização da cesariana. Ademais, mesmo quando o plano de parto é elaborado, ele apresenta um baixo grau de cumprimento^(23)^. Isso demonstra a necessidade de políticas públicas que qualifiquem e estimulem os profissionais de saúde a utilizarem esse instrumento, valorizando a autonomia da mulher^(24)^, bem como melhorando sua implementação e adesão^(23-25)^.

Os resultados integrados também mostraram que as orientações para a alta eram protocolares e, apesar do pouco espaço oferecido para o diálogo, foram compreendidas pelas mulheres e interpretadas como uma forma de comunicação eficaz. Por outro lado, faz-se necessário atualizar as práticas e romper com o poder e a autoridade resultantes do processo de institucionalização das rotinas hospitalares e da assistência horizontal e protocolar, que configura um risco à saúde perinatal^(6,26)^. A equipe de enfermagem é uma das mais envolvidas com as orientações pós-alta e, apesar dos esforços na transição do cuidado à puérpera por meio de orientações e educação para a alta, ainda são necessárias estratégias gerenciais para implementar um conjunto de ações sistematizadas que assegurem a continuidade do cuidado^(27)^, valorizem a singularidade da puérpera e sejam ofertadas de modo oportuno e integral^(28)^.

Alguns aspectos divergiram na análise integrada, o que demonstrou um ponto forte do estudo misto. Alguns achados quantitativos, inicialmente interpretados como positivos, divergiram dos resultados qualitativos. Durante as entrevistas, as mulheres explicaram que o entendimento ou o pedido para a realização dos procedimentos lhes era imposto, ou seja, elas eram apenas informadas, sem possibilidade de compartilhar das decisões. Relataram não saber o que estava acontecendo com elas, não terem a possibilidade de recusar os procedimentos e não compreenderem o motivo da indicação de cesárea. Esses aspectos fragilizaram a experiência positiva de parto, uma vez que infringiram a recomendação de comunicação efetiva entre os prestadores de cuidados da maternidade e as mulheres.

Corroborando com evidências de comunicação ineficaz, aumentam os relatos de maus-tratos no parto, por meio de intervenções não consentidas ou coerção informal, uma violação associada à etiologia da privação de autonomia, princípio central na ética médica e no cuidado em saúde^(29,30)^, e que configura uma forma de violência obstétrica^(31)^. Para alcançar melhorias na saúde materna e neonatal, bem como garantir o exercício da autonomia, é necessária uma comunicação na qual a mulher participe das decisões de forma livre e consciente^(32)^. Cabe ressaltar que a tomada de decisão da parturiente depende de informações equilibradas, completas e imparciais, exigindo do profissional de saúde maior atenção para identificar as necessidades da mulher^(33)^. No Brasil, apesar de a política de humanização do parto valorizar o protagonismo feminino, é evidente a existência de restrições ao poder de decisão da mulher no parto, em razão do biopoder, do poder disciplinar e do saber-poder profissional^(34)^.

A comunicação persuadida evidencia a complexidade das relações de poder que constituem a assistência obstétrica, sendo reproduzida também no campo do ensino. A negligência quanto ao poder decisório da mulher no parto sinaliza a necessidade de problematizar os direitos humanos das mulheres, fortalecendo-os por meio dos atores sociais envolvidos na formação, gestão e assistência^(34)^. É essencial potencializar o desenvolvimento de habilidades para o uso efetivo da comunicação, com o intuito de qualificar o cuidado obstétrico^(8)^. Portanto, faz-se necessário revisar os modelos de formação na área da saúde para promover uma mudança no cenário obstétrico, deixando de reproduzir práticas instituídas e priorizando um ensino embasado em conhecimentos científicos que coloquem a mulher como protagonista^(24)^.

Garantir o protagonismo por meio da comunicação efetiva é assegurar uma experiência positiva de parto^(35)^. Nesse sentido, este estudo chama atenção para a necessidade de fortalecer a comunicação eficaz entre profissionais de saúde e mulheres no período intraparto. É fundamental dialogar com os profissionais sobre coerção, persuasão e os direitos das mulheres no trabalho de parto. As políticas públicas podem, de fato, ser implementadas quando há uma discussão com as equipes sobre suas percepções e dificuldades em exercitar uma comunicação baseada no direito humano de ser informado de maneira clara e verdadeira^(8)^.

Convergente com a perspectiva de um modelo de comunicação eficaz, a enfermagem desempenha um papel crucial no redesenho da assistência obstétrica, promovendo uma humanização baseada em evidências^(36)^. O enfermeiro, ao fazer uso de uma boa comunicação, possibilita o empoderamento da mulher na experiência do parto^(8)^. Sua atuação nesse momento contribui para a humanização, qualificação da assistência obstétrica e consequente satisfação e segurança da parturiente. Além disso, estudos crescentes relatam a valorização da atuação do enfermeiro pelas parturientes^(37,38)^, propiciando maior bem-estar ao binômio, com menor ocorrência de intervenções e aumento do número de partos vaginais^(37)^. Tal situação evidencia o quanto o enfermeiro pode contribuir para mudanças em práticas danosas, visto que sua atuação no processo parturitivo promove maior qualidade na assistência e desafia o modelo hegemônico obstétrico^(39)^.

### Limitações do estudo

A exploração da comunicação direcionada pelo (T-IHAMC) foi uma limitação do estudo, devido à ausência de um escore nesse instrumento. A presença de um escore permitiria a conversão de dados qualitativos em quantitativos, possibilitando uma métrica para compará-los e facilitando a integração dos dados. No entanto, a utilização das duas abordagens neste estudo colaborou para superar possíveis limitações, uma vez que as insuficiências de um método puderam ser compensadas pelas potencialidades do outro, proporcionando uma perspectiva mais abrangente por meio do emprego do método misto. Ademais, o fato de os dados quantitativos terem sido coletados ainda na maternidade pode ter impactado as respostas das mulheres, que estavam sob os cuidados da equipe.

### Contribuições para a área da Enfermagem

Este artigo contribui para a enfermagem e para as equipes interdisciplinares atuantes na assistência obstétrica ao evidenciar a fragilidade da comunicação no ciclo gravídico-puerperal. Dessa forma, fortalece a reflexão sobre a necessidade de uma assistência horizontalizada, que possibilite maior autonomia à mulher. Nesse contexto, o estudo pode subsidiar políticas públicas para a efetiva implementação de instrumentos que favoreçam o exercício do poder de decisão da mulher, como o plano de parto. Ademais, espera-se que este estudo motive as equipes a avaliarem a comunicação efetiva nas maternidades como um requisito fundamental para a qualidade da assistência, seja por meio de instrumentos próprios ou auditorias internas, garantindo esse direito às parturientes.

## CONCLUSÕES

O presente estudo permitiu compreender que a comunicação entre profissionais e parturientes durante a experiência de parto envolve uma relação de poder, em que as informações são impostas, com escassas possibilidades de coparticipação da mulher, evidenciando a assimetria de poder entre profissionais, sobretudo médicos, e parturientes nas negociações, escolhas e práticas de cuidado no cenário do parto. As mulheres não recebem informações adequadas e de qualidade, tanto no pré-natal quanto durante o parto, e o plano de parto é, frequentemente, desconhecido por elas.

A análise integrada evidenciou que os procedimentos são impostos e não compreendidos, com as mulheres sendo apenas informadas da decisão do profissional, o que infringe as recomendações de comunicação eficaz conforme as diretrizes intraparto para uma experiência positiva de parto. As orientações de alta são realizadas de forma protocolar e, embora sejam compreendidas pelas mulheres e elencadas como eficazes, a reflexão que se propõe aqui é se há, de fato, uma escuta sensível, empática, respeitosa e acolhedora.

## Data Availability

Os conteúdos subjacentes ao texto da pesquisa estão contidos no manuscrito.
